# The Complex Role of Discrete Emotions in Successful Aging

**DOI:** 10.1093/geroni/igaa057.2137

**Published:** 2020-12-16

**Authors:** Jeremy Hamm, Carsten Wrosch

## Abstract

Research shows that emotions play an important role in successful aging. However, previous studies have largely focused on the implications of dimensional indicators of emotion, such as positive and negative affect. This approach may fail to capture important distinctions between discrete emotions such as sadness, loneliness, calmness, and empathy that could become more or less adaptive with age. The present studies adopt a discrete emotion perspective to examine age-related changes in the consequences of different positive and negative emotions for successful aging. Drawing from an evolutionary-functionalist perspective, Haase, Wu, Verstaen, and Levenson investigate whether sadness becomes more salient and adaptive in old age using a multi-method approach. Lee, Lay, Mahmood, Graf, and Hoppmann address the seemingly contradictory consequences of loneliness by examining how state- and trait-loneliness interact to predict older adults’ prosocial behaviors. Hamm, Wrosch, Barlow, and Kunzmann use two studies to examine the diverging salience and 10-year health consequences of discrete positive emotions posited to motivate rest and recovery (calmness) or pursuit of novelty and stimulation (excitement). Barlow and Mauss study the co-occurrence of discrete emotions and their age-dependent associations with well-being using an adult lifespan sample. Finally, Wieck, Katzorreck, Gerstorf, Schilling, Lücke, and Kunzmann examine lifespan changes in the adaptive function of empathy by assessing the extent to which empathic accuracy protects against stress-reactivity as people age. This symposium thus integrates new research on the role of discrete positive and negative emotions and will contribute to a deeper understanding of the complex relationships between emotions and successful aging.

